# In Vitro Evaluation of the Biological Effects of ACTIVA Kids BioACTIVE Restorative, Ionolux, and Riva Light Cure on Human Dental Pulp Stem Cells

**DOI:** 10.3390/ma12223694

**Published:** 2019-11-08

**Authors:** Sergio López-García, María P. Pecci-Lloret, Miguel R. Pecci-Lloret, Ricardo E. Oñate-Sánchez, David García-Bernal, Pablo Castelo-Baz, Francisco Javier Rodríguez-Lozano, Julia Guerrero-Gironés

**Affiliations:** 1 Laboratory of Cellular Therapy and Hematopoietic Transplant, Internal Medicine Department, IMIB-Virgen de la Arrixaca, University of Murcia, Avenida Buenavista s/n, 30120 Murcia, Spain; slg4850@gmail.com (S.L.-G.);; 2 Department of Genetics and Microbiology, Faculty of Biology, University of Murcia, 30100 Murcia, Spain; 3 Departament of Special Care and Gerodontology, Faculty of Medicine, University of Murcia, 30007 Murcia, Spain; mpilar.pecci@gmail.com (M.P.P.-L.); miguelr.pecci@gmail.com (M.R.P.-L.); reosan@um.es (R.E.O.-S.); juliaguerrero1@hotmail.com (J.G.-G.); 4 Endodontics Unit, Faculty of Medicine and Odontology, University of Santiago de Compostela, 15782 Santiago de Compostela, Spain; pablocastelobaz@hotmail.com

**Keywords:** dental materials, cytotoxicity, biological properties, dental pulp cells, bioactive materials

## Abstract

This study aimed to analyze the biological effects of three new bioactive materials on cell survival, migration, morphology, and attachment in vitro. ACTIVA Kids BioACTIVE Restorative (Pulpdent, Watertown, MA, USA) (Activa), Ionolux (Voco, Cuxhaven, Germany), and Riva Light Cure UV (SDI, Bayswater, Australia) (Riva) were handled and conditioned with a serum-free culture medium. Stem cells from human dental pulp (hDPSCs) were exposed to material extracts, and metabolic activity, cell migration, and cell morphology were evaluated. Cell adhesion to the different materials was analyzed by scanning electron microscopy (SEM). The chemical composition of the materials was evaluated by energy-dispersive X-ray (EDX). One-way analysis of variance followed by a Tukey test was performed (*p* < 0.05). Ionolux promoted a drastic reduction in metabolic activity and wound closure compared to the control (*p* < 0.05), whereas Activa induced adequate metabolic activity and cell migration. Moreover, SEM and immunofluorescence analysis showed abundant cells exposed to Activa. The materials showed different surface morphologies, and EDX spectra exhibited different peaks of C, O, Si, S, Ca, and F ions in glass ionomer cements. The results showed that Activa induced cell migration, cell attachment, and cell viability to a greater extent than Riva and Ionolux.

## 1. Introduction

In pediatric dentistry, several dental restorative materials have been launched on the market during recent years, including glass ionomers for clinical procedures. New formulations in glass ionomers have been proposed in order to improve matters such as handling difficulties, antibacterial properties, and anti-cariogenic effects. Furthermore, the addition of bioactive molecules in their composition is assumed to favor the release of high amounts of calcium ions, facilitating the formation of hydroxyl apatite [1,2].

The use of materials with remineralization effects is essential in deep caries with a significant risk of pulp exposure because, during carious tissue removal, bacterially contaminated and demineralized dentin is left in the pulp region and sealed beneath the restorative material, favoring remineralization of the sealed carious lesions through ion release [1,3].

Resin-based composites (RBCs) are commonly used materials due to their high mechanical properties, acceptable aesthetics, and command set. However, the monomers of these materials can spread out through the dentinal tubules to reach the pulp tissue, provoking toxic effects, such as pulpal degeneration or necrosis [4,5]. It is well-known that glass ionomer cements (GICs) can release ions such as fluoride and are less technique-sensitive than composite resins. Nevertheless, previous reports have identified a variable degree of pulp inflammation or toxicity related to their chemical composition [6,7]. Resin-modified glass ionomers (RMGIC) have been increasingly used in pediatric patients as liners or as dentine substitute materials [8,9]. These materials are considered to be very interesting in minimally invasive therapeutic restorative dentistry because they are self-adhesive, release fluoride, and are indicated in molars with molar incisor hypomineralization (MIH) [10]. Nevertheless, their poor aesthetics and unclear biocompatibility make them controversial [11].

Recently, new bioactive-based materials have been developed, such as ACTIVA Kids BioACTIVE Restorative (Activa) [2,4], a material that combines the optimal mechanical and aesthetic properties of resin materials with the ion release capacity of GICs, theoretically making it an excellent material for pediatric dentistry. It contains a blend of urethane and methacrylates with modified polyacrylic acid (44.6%); reactive glass filler (21.8%); inorganic filler (56%), patented rubberized resin (Embrace), and water. However, no information is available on its cytotoxicity.

In vitro cytotoxic or biocompatibility studies represent a suitable and reproducible method for analyzing the biological effects of new dental materials on oral cells [12]. In fact, such studies have demonstrated that unpolymerized monomers and other components from RBCs, RMGICs, and GICs can spread out though the open dentinal tubes into pulp tissue and induce cellular toxicity through 1.6 mm to 2.0 mm-thick dentin [13,14].

Thus, this study aimed to analyze the cytotoxicity of three dental restorative cements: Activa, Ionolux, and Riva Light Cure UV (Riva) on postnatal dental pulp stem cells. The null hypothesis was that the restorative materials tested would not influence the cell viability, migration, or morphology of human dental pulp stem cells (hDPSCs).

## 2. Materials and Methods

The GICs used in this study were Activa (Pulpdent, Watertown, MA, USA), Ionolux (Voco, Cuxhaven, Germany), and Riva (SDI, Bayswater, Australia). Their complete specifications can be read in Table 1.

### 2.1. Preparation of Material Extracts

GICs were prepared under aseptic conditions according to the manufacturers’ guidelines. Placed in cylinder-shaped molds of 5 mm diameter and 2.5 mm height, materials were self-cured for 20 s and then light-cured by D-Light Pro (GC, Tokyo, Japan) for 20 s, each one at 480 nm and 1400 mW/cm^2^, and stored in a CO_2_ incubator for 48 h at 37 °C to allow for full setting. Then, sample disks were sterilized with ultraviolet light for 20 min to disinfect the surface and stored in the culture medium (Dulbecco’s Modified Eagle Medium (DMEM)) at 5% CO_2_, 37 °C, and a humid atmosphere for 24 h. All procedures were carried out according to the International Organization for Standardization (ISO) guideline 10993-12 [15], and the ratio of the specimen surface area was 1.5 cm^2^/mL (ISO 10993-5) [16]. Before use, undiluted, ½, and ¼ dilutions of GICs were prepared and filtered [17].

### 2.2. Cell Culture and Characterization

The Ethical Committee approved the use of hDPSCs from seven healthy donors, 18–25 years old (University of Murcia (UM); ID: 2199/2018), at the Faculty of Medicine, University of Murcia, following Helsinki Declaration guidelines. Stem cells were obtained from the pulp chambers of extracted wisdom teeth. The molars were sectioned and the hDPSCs were obtained from root canals and the pulp chambers using a barbed nervbroach. The suspension was washed with Ca^2+^/Mg^2+^-free Hank’s balance salt solution (HBSS) (Gibco, Gaithersburg, MD, USA), and digested in 3 mg/mL collagenase-A (Gibco, Carlsbad, CA, USA) at 37 °C for 1 h. Some non-adherent cells and red blood cells were removed, washed three times with culture medium (DMEM, Invitrogen, Grand Island, NY, USA), and supplemented with 1% penicillin/streptomycin (Gibco, Carlsbad, CA, USA) and 10% foetal bovine serum (Invitrogen, Grand Island, NY, USA),). When 80% of confluence of the adherent cells was reached, they were defined as passage zero (P0). The hDPSCs were centrifuged for 5 min at 300 g and seeded in 75 cm^2^ flasks at a density of 5 × 10^3^ cells/cm^2^. Cells were subcultured once a week. They were used up to passage 4 for the experiments. The hDPSCs displayed a spindle-shaped fibroblastic morphology. Then, 1 × 10^6^ cells/tube were blocked with 0.5% bovine serum albumin (BSA) and incubated with primary antibodies targeting CD14, CD20, CD34, CD45, CD73, CD90, and CD105 (1:100, Abcam, Cambridge, MA, USA) for 1 h in the dark on ice. Stained cells were neutralized with 0.5% BSA and fixed in 2% paraformaldehyde, then analyzed with a flow cytometer (Becton Dickinson, Franklin Lakes, NJ, USA) [18].

### 2.3. Cell Metabolic Activity

An MTT assay was performed to assess the effect of different GIC extracts on hDPSCs’ metabolic activity after three days of culture. Briefly, 1 × 10^4^ hDPSCs were seeded into 96-well plates with 180 μL of DMEM for 24 h. Then, extracts of the materials were added, and cells were incubated in a 5% CO_2_ at 37 °C for 24, 48, or 72 h. Following this, 1 mg of 3-(4,5-dimethylthiazol-2-yl)-2,5- diphenyl tetrazolium bromide was dissolved in 1 mL of phosphate buffered saline (PBS) and the plates were incubated for 4 h. The medium was then aspirated and 0.2 mL of dimethyl sulfoxide (DMSO) was added to each well to solubilize the formazan crystals obtained as a result of MTT reduction by the viable cells. The absorbance of each well was detected by a plate reader (Synergy H1, BioTek, Winooski, VT, USA) at the wavelength of 570 nm (Abs570).

### 2.4. Wound Healing Assay

In vitro cell migration was assessed according to Collado-González et al. [12]. First, 1 × 10^5^ hDPSCs were seeded on 12-well plates and grown until they obtained a cell monolayer. Then, each well was scratched using a sterile 100 μL pipette tip and washed twice with PBS to remove cell debris and the detached cells. The healing process was allowed to proceed in the presence of the different material eluates, or without eluates (control group). The wound closure was evaluated at 24, 48, and 72 h. The scratch width was measured with ImageJ (National Institutes of Health, Bethesda, MD, USA) to calculate the percentage of the wound area at different times, after 24, 48, or 72 h relative to the total wound area measured at 0 h in the same well. Wound healing was evaluated separately during three periods: 0–24 h (first period), 24–48 h (second period), and 48–72 h (third period). As a way to avoid scratch width variation, the "relative wound closure" (RWC) area was calculated (RWC (%) = wound closure area, (pixel) × 100 (%)/x (pixel)).

### 2.5. Immunofluorescence Staining

After 72 h of incubation, changes in cell morphology were investigated using anti-Phalloidin (Santa Cruz Biotechnology, Santa Cruz, CA, USA) and DAPI (4,6 diamidino-2-phenylindole dihydrochloride, Sigma Aldrich, St. Louis, MO, USA, for nucleus) immunostainings. The cell-culture medium was aspirated and the cells were fixed with 4% paraformaldehyde in 0.1 M sodium phosphate buffer (PBS; pH 7.2) for 20 min at room temperature (RT). The fixed cells were permeabilized and blocked with 0.25% Triton-X-100 (Sigma Aldrich, St. Louis, MO, USA) in PBS and containing 5% (v/v) normal goat serum (NGS, Jackson Immuno Research, West Grove, PA, USA) for 1 h. CruzFluor594-conjugated Phalloidin (Santa Cruz Biotechnology, Santa Cruz, CA, USA) and DAPI (Sigma Aldrich, St. Louis, MO, USA) were used to incubate cells for F-actin and nuclei staining to visualize the actin cytoskeleton of cell nuclei. At the end of incubation for both stainings, the samples were rinsed with PBS three times, mounted with Fluoromount™ aqueous mounting medium (Sigma Aldrich, St. Louis, MO, USA), and observed under an AxioImager Zeiss light microscope (Carl Zeiss Inc., Oberkochen, Germany) equipped with an AxioCam Camera (Carl Zeiss Inc., Oberkochen, Germany). 

### 2.6. Surface Characterization of GICs and Cell Adhesion

Eighteen disks (2.5 mm height and 5 mm diameter) of the different glass ionomers were randomly divided into three groups (*n* = 6). Three of the GICs were used to visualize the cell adhesion of hDPSCs on the surface of the dental materials. A total of 5 × 10^4^ hDPSCs were seeded onto each disk and cultured for three days. Then, hDPSCs were fixed using 4% glutaraldehyde in PBS at 4 °C for 4 h. Subsequently, the samples were dehydrated in ascending concentrations of ethanol. Specimens were mounted on brass stubs and sputter-coated with gold after being placed on a copper grid for three to five minutes (Bio-RADPolaron e5400 SEM Sputter Coating System, Kennett Square, PA, USA). Finally, images were recorded at 100× and 300× magnification. Three specimens were sputter-coated with carbon and the surfaces were evaluated under an SEM (SEM Jeol 6100 EDAX, Tokyo, Japan) coupled with EDX, (EDX; Oxford INCA 350 EDX, Abingdon, UK) with operating conditions of 20 kV. The full scale for quantification was 8677 cts.

### 2.7. Statistical Analysis

The statistical analyses were performed using SPSS version 22.0 software (SPSS, Inc., Chicago, IL, USA). Each experiment was performed with three replicates. Quantitative data are presented as the mean ± standard deviation (SD). After verifying the homogeneity of variances, comparisons among groups were done using one-way analysis of variance (ANOVA) and Tukey’s multiple comparisons test to compare multivariables in more than two groups. *p* values <0.05 were considered to be significant.

## 3. Results

### 3.1. Immunophenotype of hDPSCs

**Figure 1:**
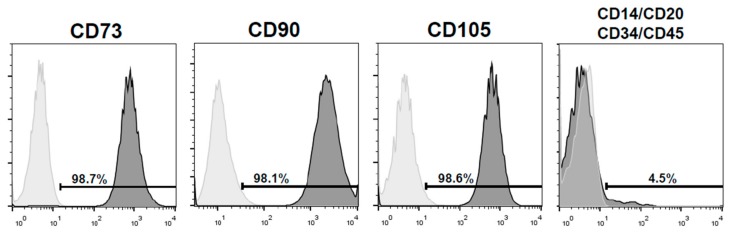
Positive expression (>95%) of mesenchymal stem cells markers CD90, CD73, and CD105, and negative expression for markers CD14, CD20, CD34, and CD45 (<5%). Control isotypes’ staining is also shown.

Dental pulp cells were spindle-shaped, consistent with the morphological characteristics of mesenchymal stem cells, and were positive for CD73 (98.7%), CD90 (98.1%), and CD105 (98.6%) and negative for CD14, CD20, CD34, and CD45 (<5%) (Figure 1).

### 3.2. Cell Metabolic Activity

**Figure 2:**
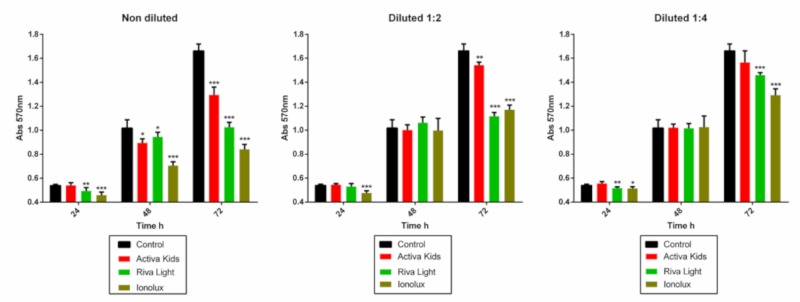
Human dental pulp stem cells’ (hDPSCs’) metabolic activity after stimulation with ACTIVA Kids BioACTIVE Restorative (Activa), Ionolux, and Riva light Cure UV (Riva), as determined by an Methyl thiazolyl tetrazolium (MTT) assay. It was performed with three replicates. Observed metabolic activity differences are shown as * *p* < 0.05; ** *p* < 0.01; *** *p* < 0.001, respectively.

The metabolic activity of hDPSCs in the presence of glass ionomer eluates was analyzed at the experimental timepoints of 24, 48, and 72 h (Figure 2). Activa did not affect metabolic activity in any sample extract in the first 24 h, while Ionolux showed significant cytotoxicity in all dilutions in the same period. Notably, no significant differences in metabolic activity were observed after incubation with the 1/2 and 1/4 dilutions of Activa, Riva, and Ionolux compared to the control group at 48 h. At 72 h, cell metabolic activity was significantly lower in the pure extract (1:1) of all materials, compared to that of the medium only (p < 0.001). However, Activa at 1:4 dilutions did not show cytotoxicity. Both Ionolux and Riva were significantly more cytotoxic than Activa in the pure extracts.

### 3.3. Wound Healing Assay

**Figure 3:**
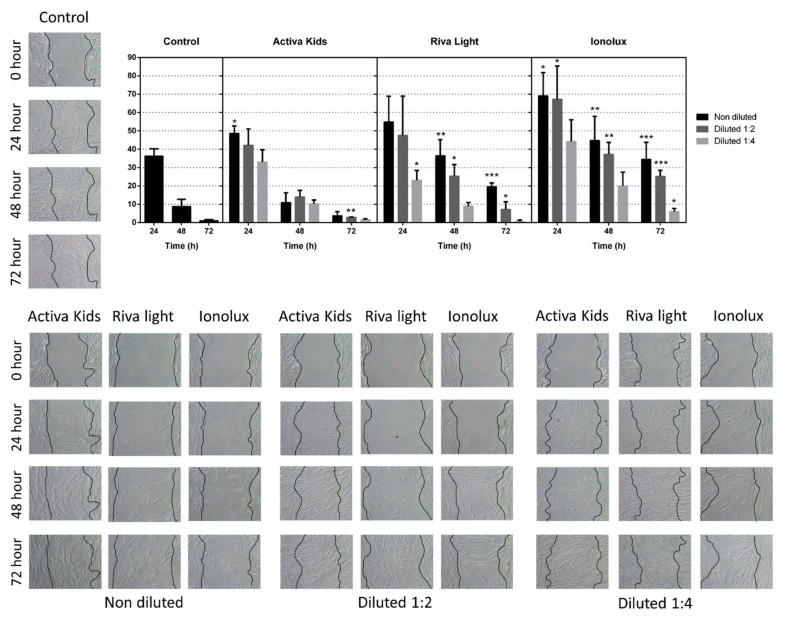
In vitro wound-healing assay. The healing process was allowed to proceed in the presence of the different material eluates, or without eluates (control group). The wound closure was evaluated at 24 h, 48 h, and 72 h. It was performed with three replicates. Data are expressed as fold of the control group (considered as 100%). Cell migration differences are shown as * *p* < 0.05; ** *p* < 0.01; *** *p* < 0.001.

The data on cell migration are shown in Figure 3. The wound healing assay has shown that cell migration was affected by Ionolux (undiluted and ½) at all the evaluated times compared to the control (*p* < 0.05). In contrast, Activa promoted cell migration, with only a slight deceleration in cells exposed to the undiluted (24 h) and ½ (72 h) dilution (*p* < 0.05). Finally, Riva revealed discrete cell migration rates, especially with undiluted and ½ dilutions (*p* < 0.05).

### 3.4. Immunofluorescence Staining

**Figure 4:**
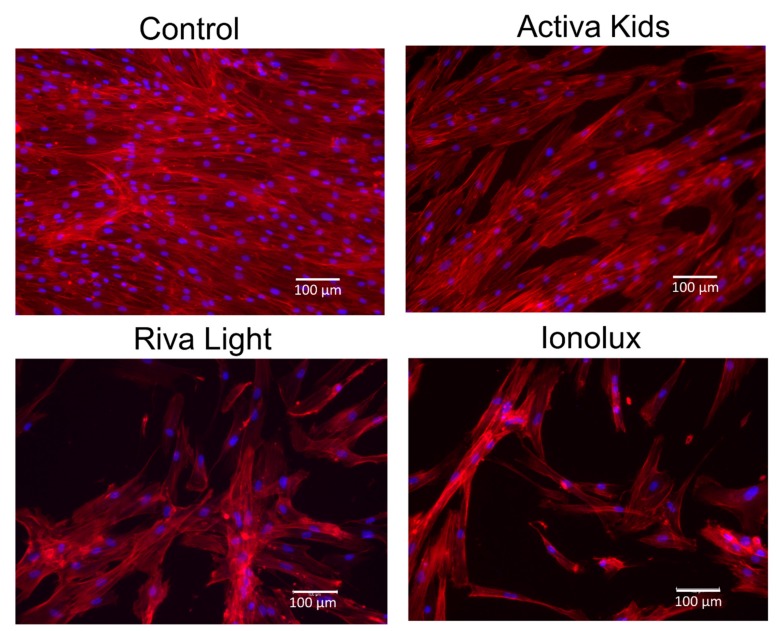
Changes in the cell morphology of hDPSCs exposed to the undiluted extracts of Activa, Ionolux, and Riva. Cytoskeleton (Phalloidin, red) and nucleus (DAPI, blue) immunostainings of hDPSCs at Day 3. The scale bar represents 100 μm for all images.

Three days following the exposure of the cell cultures to the undiluted extracts of different GICs, well-adhered and widely prominent cells were exhibited in all experimental groups (Figure 4). In general, cultures exposed to Activa exhibited higher cell density and spreading, followed by those exposed to Riva, and with the lowest cell density being observed for Ionolux.

### 3.5. Cell Adhesion on GICs and Characterization of Set Materials

**Figure 5:**
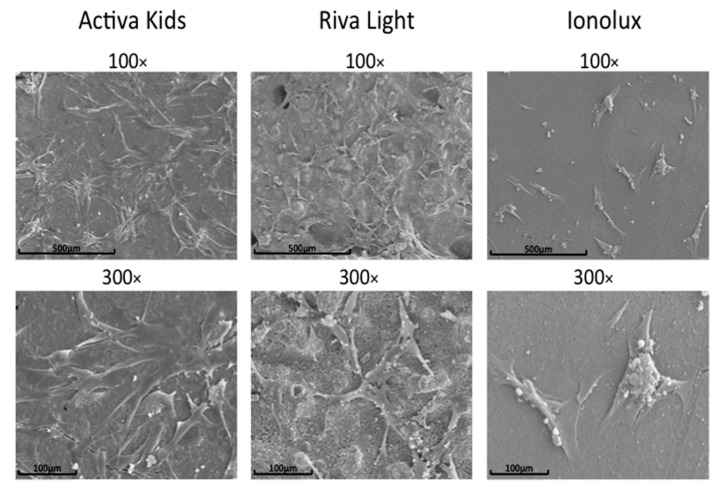
Cell attachment of hDPSCs on disks of Activa, Ionolux, and Riva at 72 h. Scale bar: 100 μm and 500 μm.

The hDPSCs cultured on Activa disks were well-adhered, with morphological characteristics of fibroblastic cells with multiple cytoplasmic extensions. In the case of Riva, less density and fewer spreading cells were detected on the surface, and finally, on the Ionolux surface, there was a drastic reduction in both density and attachment (Figure 5).

**Figure 6:**
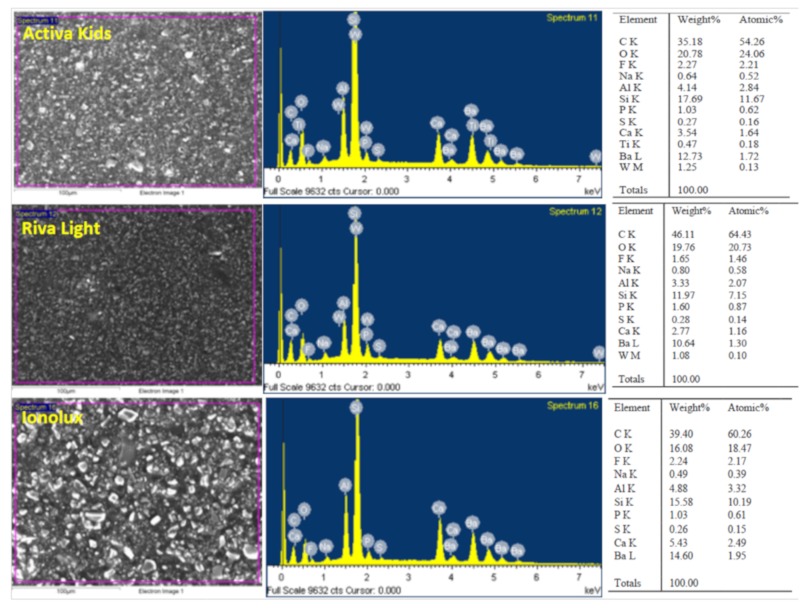
Surface properties and composition of Activa, Ionolux, and Riva under scanning electron microscopy (SEM) with energy-dispersive X-ray (EDX) analysis (SEM–EDX). SEM image (Left column), EDX spectra (Middle column), and table of elements (Right column). The scale bar represents 100 μm for all images.

The GICs analyzed showed different surface morphologies in the scanning electron microscopy analysis (Figure 6). Ionolux showed irregular crystalline structures on the surface, whereas fewer particles were found in Activa. In contrast, the surface of Riva was homogeneous with few particles. The EDX analysis gave different results for GICs in terms of percentage of weight. EDX showed peaks of carbon, oxygen, silicon, calcium, aluminum, sodium, and fluoride in the three materials, but in different percentages. No titanium was detected in Riva or Ionolux.

## 4. Discussion

The use of bioactive-based materials continues to increase, and this study analyzed the cytotoxicity of new dental restorative materials using hDPSCs. Overall, Activa promoted cell adhesion, spreading, and migration, and increased metabolic activity to a greater extent than the other tested materials.

The cytotoxicity of glass ionomers has been observed previously with several cell types such as primary human gingival fibroblasts [19], cells of the human periodontal ligament [20], osteoblast cells [13], L929 fibroblasts [21], and now hDPSCs as used in this study. A previous study detected that the cytotoxicity of some glass ionomers occurs in the organization of the F-actin cytoskeleton, which controls cell-surface motions and is essential to proliferation and differentiation [22]. The main advantage of using eluates is that they can be sterilized by filtration, allowing for the evaluation of their effects on the cells; furthermore, this method simulates a clinical situation, in which the substances spread into the pulp [23].

The standard method for evaluating the cytotoxicity or cytocompatibility of new materials is the MTT assay after one day of cell exposure (ISO 10993) [16]. Thus, higher metabolic activity was detected in cultures exposed to Activa, at both the assessed timepoints, indicating lower cytotoxicity. These results are probably related to the composition. Bioactive materials promote Ca^2+^ ion release, which has mitogenic effects on mesenchymal cells [24]. In contrast, Ionolux was cytotoxic. In agreement with other authors [25], we believe that unpolymerized HEMA is responsible for the toxicity, a finding mentioned by Bakapoulou et al. [26], who showed an evident cytotoxic effect of HEMA on dental pulp cells, which can severely disturb the odontogenic differentiation potential of HEMA, thus compromising pulp-tissue homeostasis and repair. Other reports have shown that several components such as monomers, photo-initiators, or fillers may be related to the cytotoxicity of glass ionomers [27,28,29,30]. Probably, this is the reason for low metabolic activity rates in the presence of Riva and Ionolux.

In regenerative dentistry, cell migration is an important parameter, as the migration of hDPSCs is crucial for forming a reparative dentin bridge [31]. In our study, cell migration rates with Activa were similar to those with the control group, which could be related to the positive effects of release ions on cell migration [32,33], meanwhile, with undiluted Riva and Ionolux, hDPSCs were unable to migrate in order to close the wound (*** < 0.001). Probably, the presence of unpolymerized monomers and other cytotoxic components in Riva and Ionolux provoked low cell migration rates [22].

Cell adhesion to biomaterials is essential in cell communication and interactions, and is of main importance in the process of cell differentiation [34]. In this study, the cell attachment (direct contact) and morphological characteristics of cells exposed to materials (extracts) were favorable in the case of Activa, with its high cell density and ability to spread. Among the other GIC groups, a significant reduction in cell density and cell spreading was evident in Ionolux. These findings agree with other studies reporting the cytotoxic effects of GICs [35,36].

Finally, the surface characteristics of the bioactive GICs were correlated with their biological properties [37], assessing the components of the materials by SEM and EDX analysis (SEM–EDX). The presence of calcium (Ca) was found in the three tested GICs. The release of calcium hydroxide is important because it plays a role in odontoblastic differentiation and dentin bridge formation, and in addition to that because it is responsible for the antimicrobial activity of this type of bioactive material [3,37]. Also, we detected fluoride and aluminum in all tested materials. Previous reports have shown that the aluminum content may be associated with fluoride, with which aluminum is known to complex strongly, inducing toxic effects [8]. These GICs exhibit low cytocompatibility in the freshly set state, although this decreases substantially and is time-dependent. The buffering and protein-binding effects of saliva appear to mitigate the cytotoxic effects [38,39]. On the other hand, a limitation of our study is the lack of previous studies analyzing the biological effects of Activa, Riva, and Ionolux.

Although our results are promising, to analyze the influence of other factors such as biomineralization, biocompatibility with odontoblasts, the effects of the immunological system, and the effects on pulp tissue, further studies are required.

## 5. Conclusions

The eluates from Activa displayed higher metabolic activity, cell migration, and better cell morphology than Riva and Ionolux. Activa disks showed a higher level of cell attachment compared with Riva and Ionolux.

## Figures and Tables

**Table 1:** Tested Materials.

Material	Manufacturer	Composition
**Activa Kids Restorative** (bioglass-reinforced glass ionomer restorative cement)	PULPDENT, Watertown, MA, USA	Mix of methacrylates and diurethane with modified polyacrylic acid (44.6%); reactive glass filler (21.8 wt. %); inorganic filler (56 wt. %), patented rubberized resin (Embrace), water. (wt = weight percent)
**Ionolux**	VOCO GmbH, cuxhaven, Germany	bis-GMA, polyacrilic acid, UDMA, HEMA, fluoro-alumino-silicate glass
**Riva Light Cure UV**	SDI Limited. Bayswater Victoria, Australia	Compartment 1: Acrylic acid homopolymer (15–25%), 2-hydroxyethyl methacrylate (15–25%), dimethacrylate cross-linker (10–25%), acid monomer (10–20%), tartaric acid (5–10%) Compartment 2: Glass powder (93–100%)

## References

[1] Schwendicke F., Al-Abdi A., Moscardó A.P., Cascales A.F., Sauro S. (2019). Remineralization effects of conventional and experimental ion-releasing materials in chemically or bacterially-induced dentin caries lesions. Dent. Mater..

[2] Sauro S., Makeeva I., Faus-Matoses V., Foschi F., Giovarruscio M., Maciel Pires P., Martins Moura M.E., Almeida Neves A., Faus-Llacer V. (2019). Effects of Ions-Releasing Restorative Materials on the Dentine Bonding Longevity of Modern Universal Adhesives after Load-Cycle and Prolonged Artificial Saliva Aging. Materials.

[3] Sauro S., Babbar A., Gharibi B., Feitosa V.P., Carvalho R.M., Rodrigues L.K.A., Banerjee A., Watson T. (2018). Cellular differentiation, bioactive and mechanical properties of experimental light-curing pulp protection materials. Dent. Mater..

[4] Amaireh A.I., Al-Jundi S.H., Alshraideh H.A. (2019). In vitro evaluation of microleakage in primary teeth restored with three adhesive materials: ACTIVA TM, composite resin, and resin-modified glass ionomer. Eur. Arch. Paediatr. Dent..

[5] De Caluwé T., Vercruysse C., Ladik I., Convents R., Declercq H., Martens L., Verbeeck R. (2017). Addition of bioactive glass to glass ionomer cements: Effect on the physico-chemical properties and biocompatibility. Dent. Mater..

[6] Kanjevac T., Milovanovic M., Volarevic V., Lukic M.L., Arsenijevic N., Markovic D., Zdravkovic N., Tesic Z., Lukic A. (2012). Cytotoxic effects of glass ionomer cements on human dental pulp stem cells correlate with fluoride release. Med. Chem..

[7] Geurtsen W. (2000). Biocompatibility of resin-modified filling materials. Crit. Rev. Oral Boil. Med..

[8] Sidhu S.K., Nicholson J.W. (2016). A Review of Glass-Ionomer Cements for Clinical Dentistry. J. Funct. Biomater..

[9] Kan K., Messer L., Messer H. (1997). Variability in cytotoxicity and fluoride release of resin-modified glass-ionomer cements. J. Dent. Res..

[10] De Souza J.F., Fragelli C.B., Jeremias F., Paschoal M.A.B., Santos-Pinto L., de Cassia Loiola Cordeiro R. (2017). Eighteen-month clinical performance of composite resin restorations with two different adhesive systems for molars affected by molar incisor hypomineralization. Clin. Oral Investig..

[11] Ranjkesh B., Isidor F., Kraft D.C.E., Løvschall H. (2018). In vitro cytotoxic evaluation of novel fast-setting calcium silicate cement compositions and dental materials using colorimetric methyl-thiazolyl-tetrazolium assay. J. Oral Sci..

[12] Collado-González M., García-Bernal D., Oñate-Sánchez R.E., Ortolani-Seltenerich P.S., Álvarez-Muro T., Lozano A., Forner L., Llena C., Moraleda J.M., Lozano F.J.R. (2017). Cytotoxicity and bioactivity of various pulpotomy materials on stem cells from human exfoliated primary teeth. Int. Endod. J..

[13] Michel A., Erber R., Frese C., Gehrig H., Saure D., Mente J. (2017). In vitro evaluation of different dental materials used for the treatment of extensive cervical root defects using human periodontal cells. Clin. Oral Investig..

[14] Lutfi A., Kannan T., Fazliah M., Jamaruddin M., Saidi J. (2010). Proliferative activity of cells from remaining dental pulp in response to treatment with dental materials. Aust. Dent. J..

[15] (2007). Biological evaluation of Medical Devices—Part 12: Sample Preparation and Reference Materials.

[16] (2009). Biological evaluation of Medical Devices—Part 5: Test for In Vitro Cytotoxicity.

[17] Li W., Zhou J., Xu Y. (2015). Study of the in vitro cytotoxicity testing of medical devices. Biomed. Rep..

[18] Tomás-Catalá C.J., Collado-González M., García-Bernal D., Oñate-Sánchez R.E., Forner L., Llena C., Lozano A., Castelo-Baz P., Moraleda J.M., Rodríguez-Lozano F.J. (2017). Comparative analysis of the biological effects of the endodontic bioactive cements MTA-Angelus, MTA Repair HP and NeoMTA Plus on human dental pulp stem cells. Int. Endod. J..

[19] Collado-González M., Pecci-Lloret M.R., Tomás-Catalá C.J., García-Bernal D., Oñate-Sánchez R.E., Llena C., Forner L., Rosa V., Rodríguez-Lozano F.J. (2018). Thermo-setting glass ionomer cements promote variable biological responses of human dental pulp stem cells. Dent. Mater..

[20] Celik N., Binnetoglu D., Ilday N.O., Hacimuftuoglu A., Seven N. (2019). The cytotoxic and oxidative effects of restorative materials in cultured human gingival fibroblasts. Drug Chem. Toxicol..

[21] Gupta S.K., Saxena P., Pant V.A., Pant A.B. (2013). Adhesion and biologic behavior of human periodontal fibroblast cells to resin ionomer Geristore: A comparative analysis. Dent. Traumatol..

[22] Jiang R., Lin H., Zheng G., Zhang X., Du Q., Yang M. (2017). In vitro dentin barrier cytotoxicity testing of some dental restorative materials. J. Dent..

[23] Nemoto A., Chosa N., Kyakumoto S., Yokota S., Kamo M., Noda M., Ishisaki A. (2018). Water-soluble factors eluated from surface pre-reacted glass-ionomer filler promote osteoblastic differentiation of human mesenchymal stem cells. Mol. Med. Rep..

[24] Noorani T.Y., Luddin N., Rahman I.A., Masudi S.M. (2017). In Vitro Cytotoxicity Evaluation of Novel Nano-Hydroxyapatite-Silica Incorporated Glass Ionomer Cement. J. Clin. Diagn. Res..

[25] Abidin R.M.Z., Luddin N., Omar N.S., Ahmed H.M.A. (2015). Cytotoxicity of Fast-set Conventional and Resin-modified Glass Ionomer Cement Polymerized at Different Times on SHED. J. Clin. Pediatr. Dent..

[26] Bakopoulou A., Leyhausen G., Volk J., Tsiftsoglou A., Garefis P., Koidis P., Geurtsen W. (2011). Effects of HEMA and TEDGMA on the in vitro odontogenic differentiation potential of human pulp stem/progenitor cells derived from deciduous teeth. Dent. Mater..

[27] Volk J., Ziemann C., Leyhausen G., Geurtsen W. (2009). Non-irradiated campherquinone induces DNA damage in human gingival fibroblasts. Dent. Mater..

[28] Peralta S.L., De Leles S.B., Dutra A.L., Guimarães V.B.D.S., Piva E., Lund R.G. (2018). Evaluation of physical-mechanical properties, antibacterial effect, and cytotoxicity of temporary restorative materials. J. Appl. Oral Sci..

[29] Spagnuolo G., Desiderio C., Rivieccio V., Amato M., Rossetti D.V., D’Antò V., Schweikl H., Lupi A., Rengo S., Nocca G. (2013). In vitro cellular detoxification of triethylene glycol dimethacrylate by adduct formation with N-acetylcysteine. Dent. Mater..

[30] Krifka S., Petzel C., Bolay C., Hiller K.-A., Spagnuolo G., Schmalz G., Schweikl H. (2011). Activation of stress-regulated transcription factors by triethylene glycol dimethacrylate monomer. Biomater..

[31] Sequeira D.B., Seabra C.M., Palma P.J., Cardoso A.L., Peça J., Santos J.M. (2018). Effects of a New Bioceramic Material on Human Apical Papilla Cells. J. Funct. Biomater..

[32] Calarco A., Di Salle A., Tammaro L., De Luca I., Mucerino S., Petillo O., Riccitiello F., Vittoria V., Peluso G. (2015). Long-Term Fluoride Release From Dental Resins Affects STRO-1+ Cell Behavior. J. Dent. Res..

[33] Tomson P.L., Lumley P.J., Smith A.J., Cooper P.R. (2017). Growth factor release from dentine matrix by pulp capping agents promote pulp tissue repair-associated events. Int. Endod. J..

[34] Sun Y., Liu J., Luo T., Shen Y., Zou L. (2019). Effects of two fast-setting pulp-capping materials on cell viability and osteogenic differentiation in human dental pulp stem cells: An in vitro study. Arch. Oral Boil..

[35] Subbarao C.V., Neelakantan P., Subbarao C. (2012). In vitro biocompatibility tests of glass ionomer cements impregnated with collagen or bioactive glass to fibroblasts. J. Clin. Pediatr. Dent..

[36] Zhou J., Xu Q., Fan C., Ren H., Xu S., Hu F., Wang L., Yang K., Ji Q. (2019). Characteristics of chitosan-modified glass ionomer cement and their effects on the adhesion and proliferation of human gingival fibroblasts: An in vitro study. J. Mater. Sci. Mater. Med..

[37] Farrugia C., Lung C.Y., Wismayer P.S., Arias-Moliz M.T., Camilleri J. (2018). The Relationship of Surface Characteristics and Antimicrobial Performance of Pulp Capping Materials. J. Endod..

[38] Del Angel-Mosqueda C., Hernandez-Delgadillo R., Rodriguez-Luis O.E., Ramirez-Rodriguez M.T., Munguia-Moreno S., Zavala-Alonso N.V., Solis-Soto J.M., Nakagoshi-Cepeda M.A.A., Sanchez-Najera R.I., Nakagoshi-Cepeda S.E. (2018). Hydroxyapatite decreases cytotoxicity of a glass ionomer cement by calcium fluoride uptake in vitro. J. Appl. Biomater. Funct. Mater..

[39] Schmid-Schwap M., Franz A., König F., Bristela M., Lucas T., Piehslinger E., Watts D.C., Schedle A. (2009). Cytotoxicity of four categories of dental cements. Dent. Mater..

